# Color-coded imaging of electrochromic process at single nanoparticle level†
†Electronic supplementary information (ESI) available: Dark-field microscopy setup, modification procedure, electro-chemical methods and scattering spectroscopy collection were performed as described. See DOI: 10.1039/c6sc00903d


**DOI:** 10.1039/c6sc00903d

**Published:** 2016-04-28

**Authors:** Chao Jing, Zhen Gu, Tao Xie, Yi-Tao Long

**Affiliations:** a Key Laboratory for Advanced Materials and Department of Chemistry , East China University of Science and Technology , 130 Meilong Road , Shanghai 200237 , P.R. China . Email: ytlong@ecust.edu.cn; b Physik-Department E20 , Technische Universität München , James-Franck-Str. 1 , D-85748 Garching , Germany

## Abstract

Electrochromic materials have attracted increasing attention in the field of smart devices and energy economy due to their excellent reversible chromic properties. Investigating an electrochromic process at the nano-scale is beneficial to the development of functional nano-devices exploiting chromophores. In this study, a new method for real-time imaging of an electrochromic process at the single nanoparticle level is developed based on an ultra-sensitive plasmon resonance energy transfer (PRET) technique. The scattering light intensity of nanoparticles is applied to reveal energy transfer from nanoparticles to chromophores modulated by an electrochromic reaction. This PRET-based technique achieves the detection of hundreds of molecules on the surface of a single nanoparticle. Furthermore, a color-coded amplifying method has been introduced for high-throughput, converting light intensity into easily recognized colors *via* the Matlab program. Compared with traditional electrochemical imaging techniques, this facile and rapid approach using optical techniques to characterize a real-time electrochemical process significantly enhances detection sensitivity, time and spatial resolution. Notably, the obtained electrochromic behavior of chromophores on a single nanoparticle is in good agreement with the simulated cyclic voltammetry (CV) curves on a nano-electrode. Therefore, this study provides a promising way to simultaneously monitor electrochromic reactions on single nano-electrodes with high-throughput.

## Abstract 2

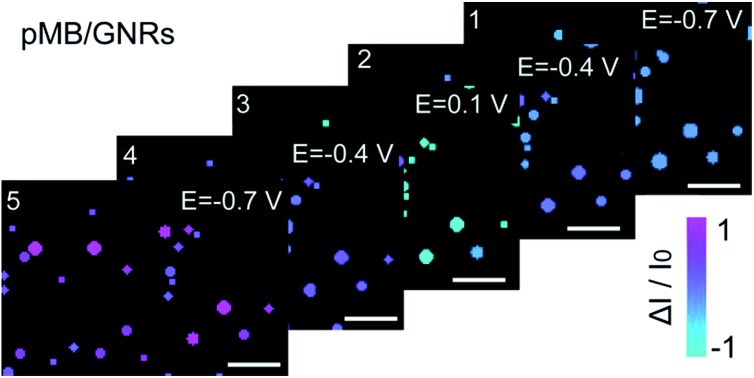
Based on a plasmon resonance energy transfer (PRET) method, the electrochromic process was imaged in real-time under potential scanning, which achieved the detection of hundreds of molecules on the surface of a single nanoparticle with high time-spatial resolution.

## Introduction

Electrochromic materials, such as transition metal oxides and organic electroactive polymers, have the capacity to alter their colors *via* redox reactions upon application of potentials.^1^,^2^ Chromophores with switchable redox states play greatly important roles in energy saving and renewable sources due to the specific absorption band change in visible and near infrared regions.^3^,^4^ Based on electrochromism, considerable smart devices and functional systems were developed to enhance energy efficiency, including that of solar heating and electronic displays.^5^,^6^ Observation of redox reactions of chromophores could provide guidance for the design of new materials. In particular, imaging electrochromic processes at the nano-scale is significant in the construction of novel devices and nanosensors.

In the past decades, electrochemical imaging techniques have attracted considerable attention for monitoring reaction information on surfaces.^7^,^8^ For instance, scanning electrochemical microscopy (SECM) enables the investigation of reaction current with high spatial resolution to micro/nano-scale, depending on the size of the electrodes.^9^–^11^ However, imaging with nano-scale resolution is mostly time-consuming for scanning large-area and difficult-to-monitor real-time processes such as cyclic voltammetry (CV) and differential pulse voltammetry (DPV) measurements. Recently, the development of optical techniques employing sensitive optical signals to reveal electrochemical reactions opened up a new way to expand electrochemical analysis down to the single nanoparticle level, particularly for fast detection.^12^,^13^ Tao used surface plasmon resonance imaging (SPRi) to observe CV processes on a single Pt nanoparticle according to the SPR signals generated by the adsorbed molecules.^14^,^15^ The combination of optical methods and electrochemistry (EC) has great potential in improving imaging sensitivity and time resolution.^16^,^17^

**Scheme 1:**
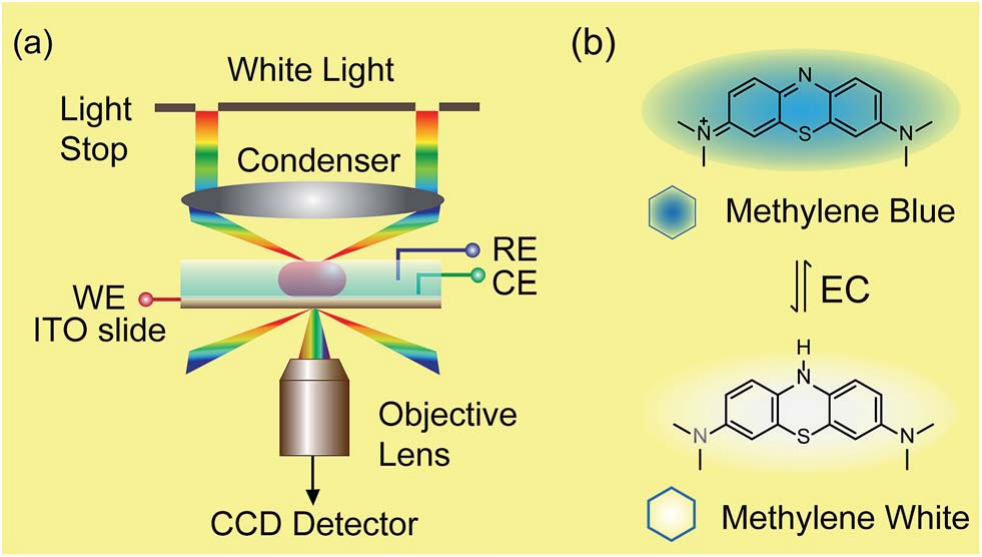
Setup of dark-field microscopy integrated with an electrochemical workstation (a) and structures of methylene blue and methylene white (b).

Notably, plasmonic nanoparticles with unique chemical, physical and optical properties have been widely applied in sensors, communication devices and energy sources.^18^–^22^ The good conductivity and abundant free surface electrons of the plasmonic particles made them excellent nano-reactors in electrochemical reactions.^23^ Thus, monitoring a redox reaction on a single nanoparticle that could act as a nano-electrode will provide detailed reaction information, eliminating the average effect of the bulk system.^24^,^25^ In 2007, plasmon resonance energy transfer (PRET) was discovered under dark-field microscopy (Scheme 1), which enhanced the sensitivity of absorption spectroscopy by several orders of magnitude, from hundreds of molecules on the surface to a single nanoparticle.^26^,^27^ When the absorption band of chromophore molecules on a particle surface is matched with the resonance scattering band of nanoparticles, resonance energy transfers from the plasmonic particles to the adsorbed molecules, leading to quenching of scattering light intensity (*I*_sca_). On the other hand, when the absorption band of chromophores has no overlap with the plasmonic scattering band, PRET does not take place, as shown in Fig. 2a–c.^28^–^31^ Therefore, it is possible to exploit the *I*_sca_ of nanoparticles to detect the electrochromic process of chromophores with ultra-high sensitivity, benefitting from the PRET phenomenon.

Herein, we demonstrate a novel method to real-time image the electrochromic process of chromophores under CV scanning at the single nanoparticle level based on PRET. In this study, methylene blue (MB) was selected as the typical analyte for its excellent electrochemical and optical properties (Scheme 1).^32^–^34^ MB with blue color could be reduced to colorless methylene white (MW) *via* an electrochromic reaction, which changes the absorption band of the molecules and induces PRET alternation. Using the scattering intensity of plasmonic nanoparticles to observe the electrochemical reaction enables rapid imaging at the single nanoparticle level with high spatial resolution and high throughput. We believe that it is a promising approach for the *in situ* detection of a reaction process on a nano-electrode, which is meaningful for the development of nano-electrochromic devices.

## Results and discussion

**Fig. 1:**
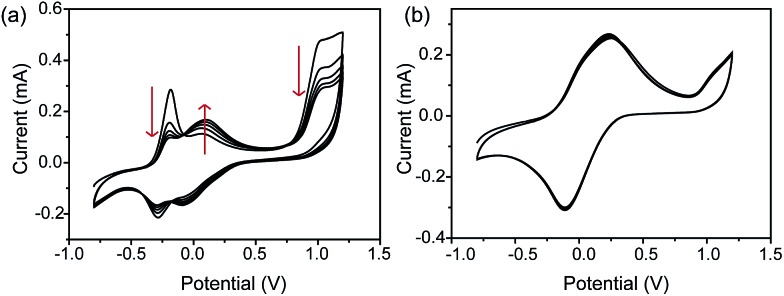
Cyclic voltammograms (CVs) of electrochemical polymerization process in 0.1 mM methylene blue solution on a GNRs modified ITO electrode, at the beginning (a) and after scanning 100 cycles (b) in the range from –0.80 V to 1.20 V. Electrolyte: 0.1 M PBS solution (pH = 7.0). Scan rate: 100 mV s^–1^, *vs.* saturated calomel electrode (SCE).

**Fig. 2:**
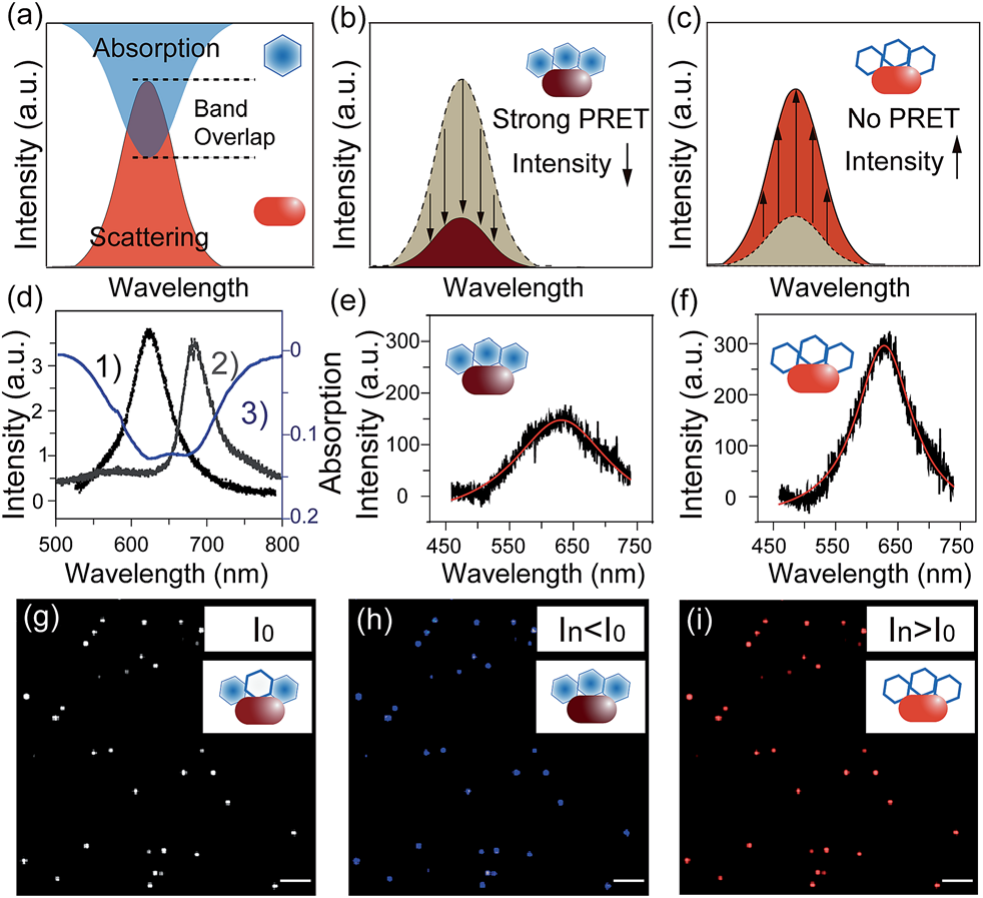
(a–c) Scheme of PRET between the gold nanorod and MB/MW inducing the change of scattering light intensity. (d) Scattering spectrum of a single gold nanorod (1), fluorescence spectrum of pMB (2) and absorption spectrum of pMB (3). (e and f) Scattering spectra of a single nanorod modified with pMB (e) and pMW (f). (g–i) Scheme of calculation results for scattering light intensity decreasing (blue) and increasing (red) modulated by the PRET effect in (e) and (f); scale bars in (g–i) are 10 μm.

**Fig. 3:**
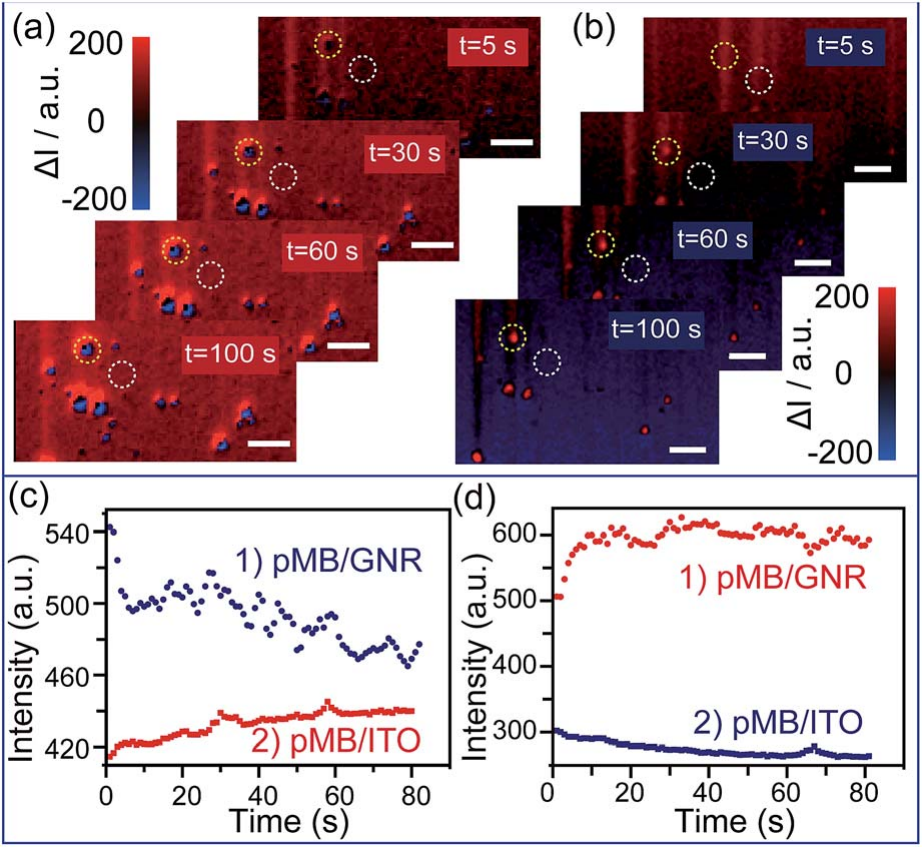
(a) Time dependent high-throughput scattering intensity change of mono-dispersed pMB/GNRs under potential of 0.10 V, *vs.* Pt quasi-reference, in 0.1 M PBS (pH = 7.0) solution, calculated by Matlab program. (b) Time dependent high-throughput scattering intensity change of mono-dispersed pMB/GNRs under the potential of –0.70 V calculated by Matlab program. (c) Scattering intensity change (1) of single pMB/GNR particle labeled in (a) (yellow circles) and pMB/ITO background intensity change (2) labelled in (a) (white circles) under the potential of 0.10 V. (d) Scattering intensity change (1) of single pMB/GNR particle labeled in (b) (yellow circles) and pMB/ITO background intensity change (2) labelled in (b) (white circles) under the potential of –0.70 V. Scale bars are 10 μm.

Dark-field microscopy combined with electrochemistry work station is shown in Scheme 1a. Gold nanoparticles were modified on an ITO slide which acted as a working electrode. A three-electrode system was built in which two Pt wires functioned as quasi-reference and counter electrodes. The potential of the Pt quasi-reference electrode was calibrated *via* potassium ferricyanide solution (Fig. S1, ESI†). As PRET takes place on the surface of a nanoparticle, MB was modified on a GNR/ITO substrate by electrochemical polymerization, as shown in Fig. 1. In 0.1 M PBS (pH = 7.0) solution containing 0.1 mM MB, CV scanning from –0.80 V to 1.20 V at a scan rate of 100 mV s^–1^ was implemented. At the beginning, it was obvious that the CV curves exhibited an oxidization peak at –0.18 V and a reduction peak at –0.28 V owing to MB molecules, and a polymerization peak current also occurred at 1.02 V. After scanning for 100 cycles, the peak current of MB disappeared and the reversible redox of polymerized MB/MW (pMB/pMW) became dominant. After rinsing with water to remove the free MB molecules on the ITO surface, the pMB modified GNR/ITO was used in the imaging process. Polymerized MB has a broad absorption band from 550 nm to 750 nm (Fig. 2d-3). As pMB has a fluorescence band from 650 nm to 720 nm (Fig. 2d-2), gold nanorods (GNR) with a scattering band of 600–650 nm were selected as nanoprobes to both meet the condition of PRET (Fig. 2a–c) and avoid the influence of fluorescence. SEM images of the mono-dispersed GNRs are displayed in Fig. S2 (ESI†). From Fig. 2e, the scattering spectrum of pMB/GNR showed weak intensity because of PRET. After reducing pMB to pMW by electrochemistry, the GNR exhibited obvious intensity enhancement, suggesting that PRET induced the intensity change of scattering light, which revealed the electrochemically controlled pMB/pMW states (Fig. 2f). In addition, scattering spectra of GNR in MB solution modulated by a chemical reaction showed a similar spectral change (Fig. S3, ESI†). Therefore, based on the controllable PRET, we developed a facile and easy method to image the electrochromic process of chromophores according to the scattering intensity of single nanoparticles. In order to image the electrochemical process with high throughput, a rapid color-coded calculation method was introduced. The dark-field image of mono-dispersed gold nanoparticles is showed in Fig. 2g. The brightness represents the scattering light intensity of each nanoparticle. We indicated the intensity change with visible colors using the Matlab program. Increase of intensity was displayed as red color and decreased intensity was displayed as blue color, as illustrated in Fig. 2h and i. Therefore, the electrochemical process could be easily monitored *via* the color change of the scattering spots of every single nanoparticle. It can be noted that at the beginning, both pMB and pMW existed on the surface of GNR due to the long-time electrochemical scanning for polymerization. In addition, the fluorescence of pMB was used as contrast to support the experimental results obtained from PRET.

Based on previous reports, the scattering spectral intensity of GNR is also affected by the applied potentials due to the surface electron density change.^35^ Upon applying a positive potential, the nanoparticles showed spectral intensity decrease for the discharging of surface electron loss. When a negative potential was applied, the scattering spectra exhibited increased intensity because of the electron charging. To investigate the electron density effect on scattering intensity of GNRs, control experiments were implemented, as shown in Fig. S4.† Under the potential from –0.70 V to 0.10 V, the scattering spectral peak intensity *λ*_max_ of the pMB/GNR showed much more obvious change than the *λ*_max_ of a bare GNR. This result demonstrated that the effect of surface electron density could be ignored, as the PRET process is the primary factor determining the spectral change. The results certified the practicability of using PRET-based scattering intensity to monitor the electrochemical process on a single nanoparticle.

To achieve the detection, a background image of the nanoparticles was firstly recorded under an open circuit. During the potential scanning, the intensity of each pixel in the captured images was subtracted by the intensity of the corresponding pixel in the background image. The differences are shown as blue and red colors. The electrochromic process of pMB under a constant potential of 0.10 V is depicted in Fig. 3a. It is obvious that during application of the potential of 0.10 V, GNRs exhibited blue colors, indicating the gradually decreased scattering intensity. This result was attributed to the fact that the increasing amount of pMB enhanced the PRET effect, which induced the resonance energy loss of particles. In contrast, the pMB/ITO background became red, suggesting intensity increase due to the enlarged fluorescence intensity of pMB. The fluorescence enlargement proved that the electrochemical oxidation process occurred and supported the imaging results. Fig. 3c shows the light intensity change of a single gold nanorod modified with pMB (a) the and pMB/ITO background (b) under a potential of 0.10 V. Notably, the altering of light intensity exhibited similar trends of typical amperometric *I*_c_–*t* curves, which demonstrated that this method could be applied to the detection of an electrochemical process successfully. As expected, the scattering and fluorescence intensity both differed under the potential of –0.70 V, showing the opposite color. The scattering light of pMB/GNR turned red and the fluorescence of pMB/ITO became blue, as displayed in Fig. 3b and d.

**Fig. 4:**
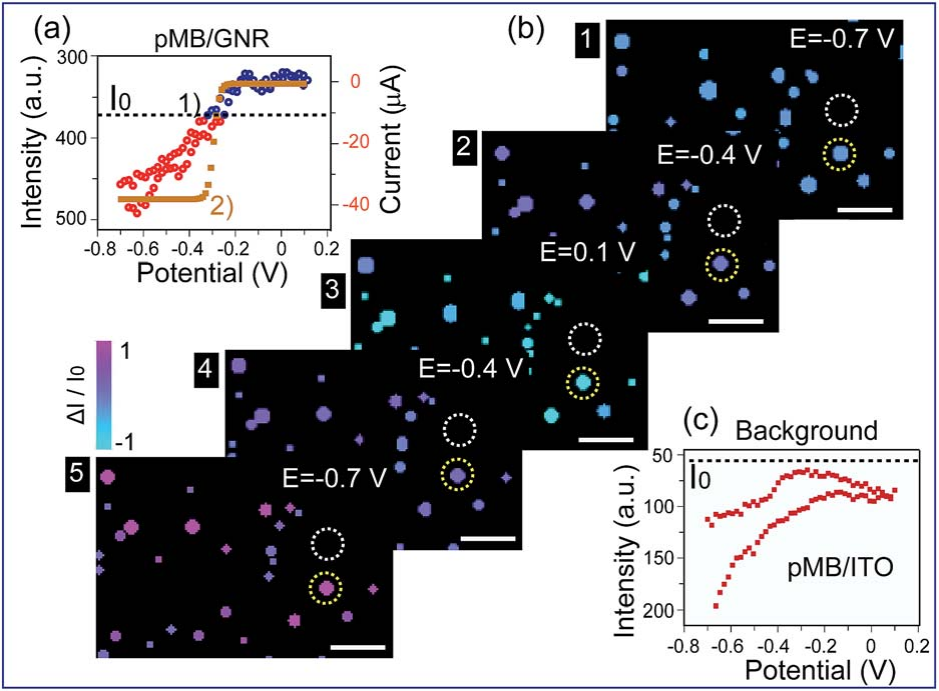
(a) (1) Scattering intensity change of a single gold nanorod labelled in (b) (yellow circles) under cyclic voltammetry (CV) scanning, at the range of –0.70 V to 0.10 V, scan rate of 10 mV s^–1^, *vs.* Pt quasi-reference, in 0.1 M PBS (pH = 7.0) solution. (2) Simulated CV curve of pMB on a 100 nm electrode. (b) Time dependent high-throughput light intensity change of mono-dispersed pMB/GNRs under CV scanning calculated by Matlab program. (c) PMB/ITO background intensity change labelled in (b) (white circles) under CV scanning. Scale bars are 10 μm.

Furthermore, this method was applied to real-time imaging of the CV scanning process of pMB molecules at the single nanoparticle level. CV scanning was carried out from –0.70 V to 0.10 V in PBS solution with scan rate of 10 mV s^–1^. The capture time of a dark-field image is 2 s. It could be noticed that the imaging process in Fig. 3 showed “halo” features. This phenomenon was because of the slight position shift of the sample stage during the electrochemical scanning. To avoid the “halo” effect and the influence of fluorescence background (imaging results based on previous method were displayed in Fig. S5 in the ESI†), we improved the imaging method by introducing a particle recognition system through the Matlab program. Meanwhile, a new color bar from blue to purple was used to better exhibit the intensity change of nanoparticles, as shown in Fig. 4b. From Fig. 4b-1, no obvious change was observed at the beginning. Then, the intensity of gold nanoparticles increased slowly along with time due to the applied negative potential (–0.70 V to –0.30 V) reducing pMB into pMW. While it increased to the oxidation potential of pMW (–0.30 V to 0.10 V), the color of the nanoparticles started to turn blue, indicating the production of pMB, which enhanced the PRET effect. It is worthy to note that the intensity decrease started from a negative potential of *ca.* –0.30 V, which proved that the primary effect was PRET rather than electron density change. As the potential was continuously decreased during the cathodic back scan, the intensity of nanoparticles increased gradually because of the reduction of pMB molecules. When the potential reached –0.70 V, the nanoparticles clearly displayed a purple color. The light intensity of the pMB/GNRs showed cyclic changes during CV scanning. However, the fluorescence of pMB molecules on the ITO substrate showed increased intensity constantly without reversible intensity change, as shown in Fig. S5.† Moreover, for a single gold nanorod, the scattering intensity (Fig. 4a-1) exhibited reversible altering, which is in good agreement with the simulated CV curve on a nano-electrode (Fig. 4a-2).^36^ The intensity change of fluorescence in the background increased gradually after long-time scanning (Fig. 4c). These results suggested that the electrochromic process could only be modulated effectively for hundreds of pMB molecules on a single nanorod surface. Nevertheless, for the large amount of pMB molecules on the ITO slide, the background showed irreversible intensity increase due to the low electron transfer efficiency of the redox reaction. The distinct intensity differences between pMB/GNR and pMB/ITO confirmed that the PRET-based imaging method improved the sensitivity dramatically, which achieved the detection of hundreds of molecules on a particle surface with high throughput. Moreover, it allows us to readily monitor real-time CV scanning and provides a novel approach with high spatial resolution to reveal electrochromic properties on a “nano-electrode”.

## Conclusions

In conclusion, we developed a new method for real-time imaging of an electrochromic process on single nanoparticles based on a PRET technique with high spatial resolution. This ultra-sensitive approach achieved the observation of hundreds of molecules on a single nanoparticle surface. Moreover, it avoided the time-consuming and high-cost limitations of conventional electrochemical methods such as SECM and enabled the monitoring of continuous potential scanning. Importantly, the introduction of a color-coded amplifying program afforded visible and high-throughput detection of electrochromic reactions. Furthermore, for different chromophore molecules, nanoparticles with various morphologies and compositions could be selected to match their absorption bands. Therefore, this facile and fast opto-electronic approach has promising applications in the detection of electrochromic processes, particularly for analysis on the nanoscale, which could improve the understanding of reaction mechanisms and expand the applications of chromic materials in molecular devices and nanosensors.

## Supplementary Material

Supplementary information

## References

[cit1] Gunbas G., Toppare L. (2012). Chem. Commun..

[cit2] Li C., Bai H., Shi G. (2009). Chem. Soc. Rev..

[cit3] Thakur V. K., Ding G., Ma J., Lee P. S., Lu X. (2012). Adv. Mater..

[cit4] Cai G., Cui M., Kumar V., Darmawan P., Wang J., Wang X., Lee-Sie Eh A., Qian K., Lee P. S. (2016). Chem. Sci..

[cit5] Wade C. R., Li M., Dinca M. (2013). Angew. Chem., Int. Ed..

[cit6] Cui B.-B., Yao C.-J., Yao J., Zhong Y.-W. (2014). Chem. Sci..

[cit7] Lai S. C., Patel A. N., McKelvey K., Unwin P. R. (2012). Angew. Chem., Int. Ed..

[cit8] O'Connell M. A., Lewis J. R., Wain A. J. (2015). Chem. Commun..

[cit9] Kueng A., Kranz C., Lugstein A., Bertagnolli E., Mizaikoff B. (2003). Angew. Chem., Int. Ed..

[cit10] Kranz C. (2014). Analyst.

[cit11] Li M. S., Filice F. P., Ding Z. (2014). J. Inorg. Biochem..

[cit12] Fang Y. M., Wang W., Wo X., Luo Y. S., Yin S. W., Wang Y. X., Shan X. N., Tao N. J. (2014). J. Am. Chem. Soc..

[cit13] Hill C. M., Pan S. L. (2013). J. Am. Chem. Soc..

[cit14] Shan X., Patel U., Wang S., Iglesias R., Tao N. (2010). Science.

[cit15] Shan X., Díez-Pérez I., Wang L., Wiktor P., Gu Y., Zhang L., Wang W., Lu J., Wang S., Gong Q., Li J., Tao N. (2012). Nat. Nanotechnol..

[cit16] Oja S. M., Fan Y., Armstrong C. M., Defnet P., Zhang B. (2016). Anal. Chem..

[cit17] Ozbay E. (2006). Science.

[cit18] Zheng X., Liu Q., Jing C., Li Y., Li D., Luo W., Wen Y., He Y., Huang Q., Long Y.-T., Fan C. (2011). Angew. Chem., Int. Ed..

[cit19] Raimondi F., Scherer G. G., Kotz R., Wokaun A. (2005). Angew. Chem., Int. Ed..

[cit20] Xiao L., Wei L., Cheng X., He Y., Yeung E. S. (2011). Anal. Chem..

[cit21] Zhang L., Chen H., Wang J., Li Y. F., Wang J., Sang Y., Xiao S. J., Zhan L., Huang C. Z. (2010). Small.

[cit22] Février M., Gogol P., Aassime A., Mégy R., Delacour C., Chelnokov A., Apuzzo A., Blaize S., Lourtioz J.-M., Dagens B. (2012). Nano Lett..

[cit23] Luo X., Morrin A., Killard A. J., Smyth M. R. (2006). Electroanalysis.

[cit24] Jing C., Rawson F. J., Zhou H., Shi X., Li W. H., Li D. W., Long Y. T. (2014). Anal. Chem..

[cit25] Zhou H., Liu Q., Rawson F. J., Ma W., Li D. W., Li D., Long Y. T. (2015). Chem. Commun..

[cit26] Liu G. L., Long Y. T., Choi Y., Kang T., Lee L. P. (2007). Nat. Methods.

[cit27] Choi Y., Park Y., Kang T., Lee L. P. (2009). Nat. Nanotechnol..

[cit28] Choi Y., Kang T., Lee L. P. (2008). Nano Lett..

[cit29] Cushing S. K., Li J. T., Meng F., Senty T. R., Suri S., Zhi M. J., Li M., Bristow A. D., Wu N. Q. (2012). J. Am. Chem. Soc..

[cit30] Qu W.-G., Deng B., Zhong S.-L., Shi H.-Y., Wang S.-S., Xu A.-W. (2011). Chem. Commun..

[cit31] Shi L., Jing C., Gu Z., Long Y.-T. (2015). Sci. Rep..

[cit32] Erdem A., Kerman K., Meric B., Ozsoz M. (2001). Electroanalysis.

[cit33] Krpetić ž., Singh I., Su W., Guerrini L., Faulds K., Burley G. A., Graham D. (2012). J. Am. Chem. Soc..

[cit34] Zhang H., Zhai Y., Dong S. (2014). Talanta.

[cit35] Novo C., Funston A. M., Gooding A. K., Mulvaney P. (2009). J. Am. Chem. Soc..

[cit36] Arrigan D. W. (2004). Analyst.

