# Supplementary material for: Color-coded imaging of electrochromic process at single nanoparticle level
Source: Chem Sci. 2016 Apr 28;7(8):5347–51. doi: 10.1039/c6sc00903d (PMC6020751; doi:10.1039/c6sc00903d)
Supplement: Supplementary file 1 [file SC-007-C6SC00903D-s001.pdf]

Electronic Supplementary Material (ESI) for Chemical Science.
This journal is © The Royal Society of Chemistry 2016

Supporting Information

Color-coded Imaging of Electrochromic Process at Single Nanoparticle Level

Chao Jing,[a,b] Zhen Gu,[a] Tao Xie,[a] and Yi-Tao Long*[a]

[a] Key Laboratory for Advanced Materials and Department of Chemistry, East China University of Science and Technology, Shanghai 200237, P.R. CHINA

[b] Physik-Department E20, Technische Universität München, James-Franck-Str. 1, D-85748 Garching, Germany

Corresponding Author

ytlong@ecust.edu.cn

EXPERIMENTAL SECTION

Materials. All reagents were of analytical grade. Absolute ethanol (AR), acetone (AR), methylene blue, potassium nitrate (AR) were purchased from sigma (USA). Gold nanorods (40 nm × 65 nm, 10 O.D.) were purchased from Nanoseedz (Hong Kong, China). Ultrapure water with a resistivity of 18.2 MΩ·cm was produced using a Milli-Q apparatus (Millipore, USA) and used in the preparation of all the solutions. The indium tin oxide (ITO) slides were purchased from Geao Co. Ltd. (Wuhan, China).

Preparation of the Samples. Gold nanoparticles used in the experiments were immobilized on the (ITO) slides. The surfaces of ITO slides were cleaned in an ultrasonic bath. The slides were first ultrasonic treated in ethanol and acetone to remove oily matter and then treated in water to remove water-soluble matter. The samples were cleaned in each solvent for more than 1 h. The cleaned ITO slides were then modified with GNPs via electrostatic adsorption by placing them in the diluted gold colloid solution (50 times) for 15 min. The GNP-functionalized ITO slides were rinsed with water and dried under a stream of ultrapure nitrogen prior to the dark-field measurements. The nanoparticles adsorbed on ITO slides were stable during the electrochemical scanning as shown in Figure S6 in the Supporting Information.

Dark-Field Microscopy and Scattering Spectroscopy. The dark-field measurements were carried out on an inverted microscope (eclipse Ti-U, Nikon, Japan) that was equipped with a dark-field condenser (0.8 < NA < 0.95) and a 40× objective lens (NA = 0.8). The white light source was a 100 W halogen lamp. The GNP-functionalized slides were immobilized on a platform, and the white light source was used to excite the GNPs and generate plasmon resonance scattering light. A true-color digital camera (Nikon DS-fi, Japan) was used to capture the dark-field color images. The scattering light of gold nanoparticle was split by a monochromator (Acton SP2300i, Princeton Instruments, USA) that was equipped with a grating (grating density: 300 lines/mm; blazed wavelength: 500 nm) and recorded by a spectrometer CCD (Pixis 400, Princeton Instruments, USA) to obtain the scattering spectra. The exposure time for every scattering spectrum was 10 s.

Electrochemistry. All electrochemical measurements were performed using a CHI 660c (Chenhua, Shanghai, China) electrochemistry station. Here a homemade micro electrochemical cell has been developed, the ITO slide acts as the working electrode, two Pt wires function as the counter and reference electrode, respectively. Here the Pt

wire was selected as reference electrode to eliminate the influence of Cl^- ions dissociated from AgCl. All measurements were performed at 25 °C.

Calibration of Pt Reference Electrode. The potential of Pt electrode was calibrated via potassiumferricyanide as shown in Supplementary Figure S1. The $E_0^{1/2}$ of 1.00 mM $K_3[Fe(CN)_6]$ solution using Pt quasi-reference electrode and saturated calomel electrode (SCE) was -0.127 V and 0.211 V, respectively.

Modification of Polymerized MB (pMB). PMB was modified on GNR/ITO substrate via CV scanning from -0.80 V to 1.20 V with a scan rate of 100 mV/s in 0.1 M PBS (pH=7.0) solution containing 0.1 mM MB, *vs.* saturated calomel electrode (SCE). At first, it is obvious that CV curves exhibited an oxidization peak at -0.18 V and a reduction peak at -0.28 V owing to MB molecules, and also a polymerization current peak was occurred at 1.02 V. Through scanning for 100 circles, the peak current of MB disappeared and the reversible redox of pMB/pMW was dominant. After rinsed with water to remove the free MB molecules on ITO surface, the pMB modified GNR/ITO was applied in the imaging process based on PRET technique.

SUPPLEMENTARY EXPERIMENTS

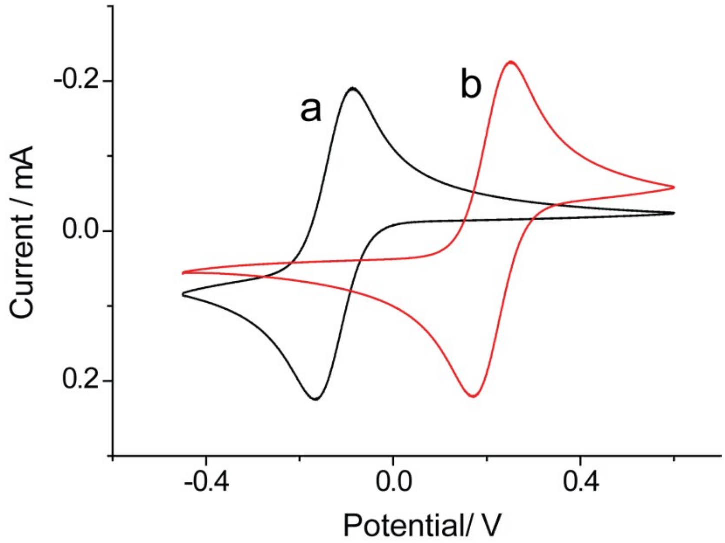

Figure S1 Cyclic voltammograms (CVs) of 1 mM K_3 $[Fe(CN)_6]$ solution on bare ITO electrode. Electrolyte: 0.1 M KNO_3 solution. Scan rate: 100 mV/s, vs. quasi-reference electrode (a) and saturated calomel electrode (SCE) (b).

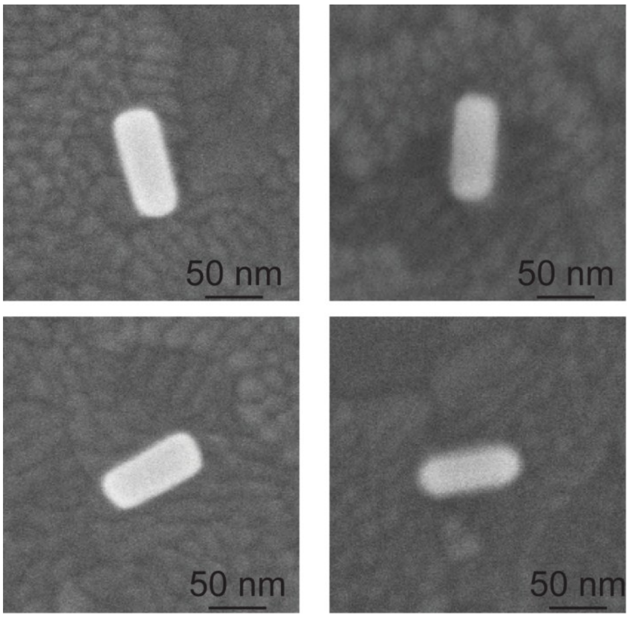

Figure S2 SEM images of mono-dispersed gold nanorods.

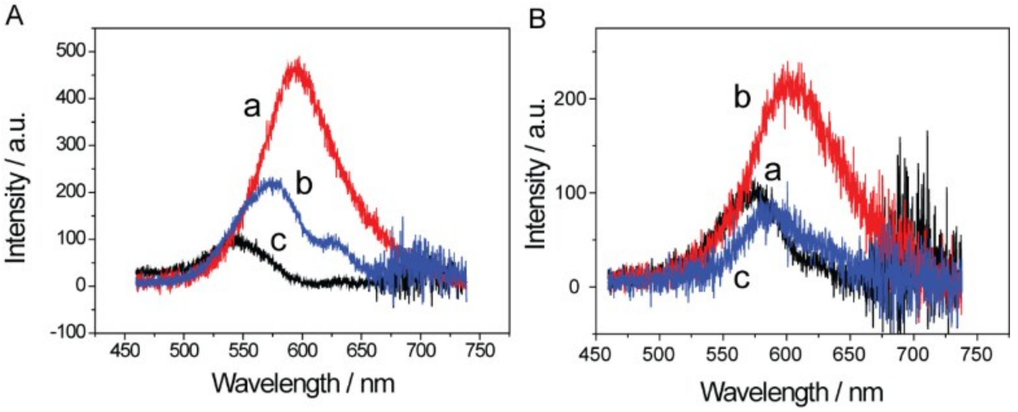

Figure S3 (A) Scattering spectra of single gold nanorod before (a) and (b-c) after the addition of methylene blue with different concentrations: (b) 10 nM and (c) 100 nM. (B) Scattering spectra of single gold nanorod after the addition of different solutions: (a) 100 nM methylene blue and 0.1 mM glucose; (b) 100 nM methylene blue, 0.1 mM glucose and 0.1 mM NaOH solution; (c) 100 nM methylene blue, 0.1 mM glucose and 0.1 mM H_2SO_4 solution.

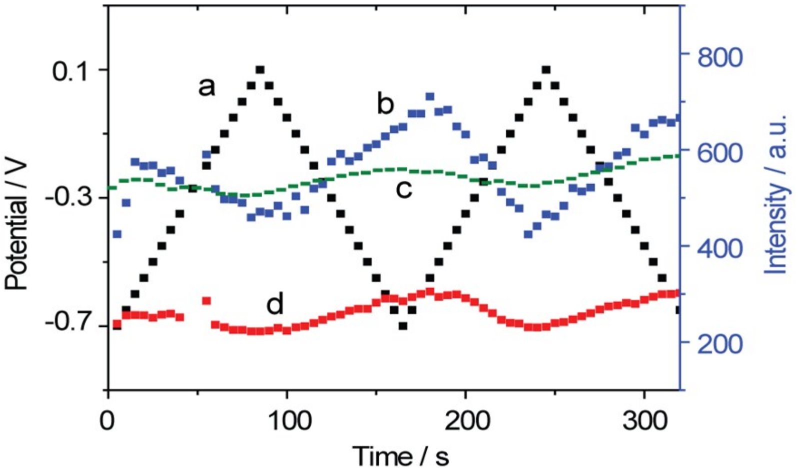

Figure S4 Scattering spectra intensity change of single gold nanorod under applied potential (a) from -0.7 V to 0.1 V with scan rate of 10 mV/s vs. Pt quasi-reference: (b) Scattering spectra intensity change at peak wavelength of single gold nanorod with Poly-MB; (c) Scattering spectra intensity change at peak wavelength of bare single gold nanorod; (d) Scattering spectra intensity change at entire wavelength range of single gold nanorod with Poly-MB.

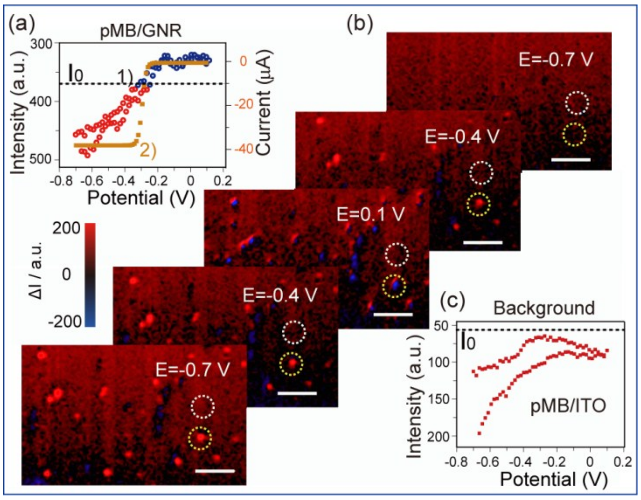

Figure S5 (a) (1) Scattering intensity change of a single gold nanorod labelled in (b) (yellow circles) under cyclic voltammetry (CV) scanning, at the range of -0.70 V to 0.10 V, scan rate: 10 mV/s, vs. Pt quasi-reference, in 0.1 M PBS (pH=7.0) solution. (2) Simulated CV curve of pMB on a 100 nm electrode. (b) Time dependent high throughput light intensity change of monodispersed gold nanorods under CV scanning calculated by Matlab. (c) pMB/ITO background intensity change labelled in (b) (white circles) under CV scanning. Scale bars are 10 μm.

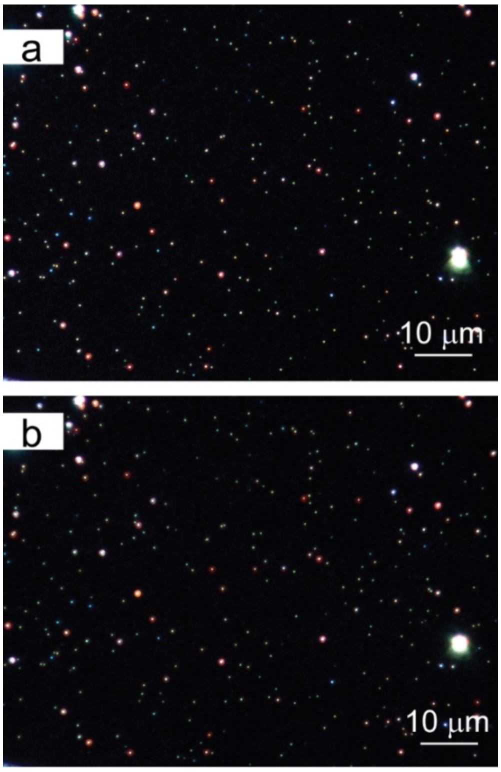

Figure S6 Dark-field images of gold nanoparticles before (**a**) and after (**b**) CV scanning from 0.10 V to 1.0 V for 36 circles.
